# Protein phosphatase methylesterase‐1 (PME‐1) expression predicts a favorable clinical outcome in colorectal cancer

**DOI:** 10.1002/cam4.541

**Published:** 2015-09-17

**Authors:** Amanpreet Kaur, Adam Elzagheid, Eva‐Maria Birkman, Tuulia Avoranta, Ville Kytölä, Eija Korkeila, Kari Syrjänen, Jukka Westermarck, Jari Sundström

**Affiliations:** ^1^ Department of PathologyUniversity of TurkuTurku20520Finland; ^2^ Turku Centre for BiotechnologyUniversity of Turku and Åbo Akademi UniversityTurku20520Finland; ^3^ TuBS and TuDMM Doctoral ProgrammesTurku20520Finland; ^4^ Department of PathologyFaculty of MedicineBenghazi UniversityPO Box 1308BenghaziLibya; ^5^ Biotechnology Research CenterTripoliLibya; ^6^ Department of Oncology and RadiotherapyUniversity of Turku and Turku University HospitalTurku20521Finland; ^7^ Department of Social Services and HealthcareCity of HelsinkiHelsinki00099Finland; ^8^ BioMediTechUniversity of TampereTampere33520Finland; ^9^ Department of Clinical ResearchBiohit OyjHelsinki00880Finland; ^10^ Molecular Oncology Research CenterBarretos Cancer HospitalBarretos14784‐400Brazil

**Keywords:** Biomarker, colorectal cancer, PME‐1, PP2A, survival, TCGA

## Abstract

Colorectal cancer (CRC) accounts for high mortality. So far, there is lack of markers capable of predicting which patients are at risk of aggressive course of the disease. Protein phosphatase‐2A (PP2A) inhibitor proteins have recently gained interest as markers of more aggressive disease in certain cancers. Here, we report the role of PP2A inhibitor PME‐1 in CRC. PME‐1 expression was assessed from a rectal cancer patient cohort by immunohistochemistry, and correlations were performed for various clinicopathological variables and patient survival. Rectal cancer patients with higher cytoplasmic PME‐1 protein expression (above median) had less recurrences (*P* = 0.003, *n* = 195) and better disease‐free survival (DFS) than the patients with low cytoplasmic PME‐1 protein expression (below median). Analysis of *PPME‐1* mRNA expression from TCGA dataset of colon and rectal adenocarcinoma (COADREAD) patient cohort confirmed high *PPME1* expression as an independent protective factor predicting favorable overall survival (OS) (*P* = 0.005, *n* = 396) compared to patients with low *PPME1* expression. CRC cell lines were used to study the effect of PME‐1 knockdown by siRNA on cell survival. Contrary to other cancer types, PME‐1 inhibition in CRC cell lines did not reduce the viability of cells or the expression of active phosphorylated AKT and ERK proteins. In conclusion, PME‐1 expression predicts for a favorable outcome of CRC patients. The unexpected role of PME‐1 in CRC in contrast with the oncogenic role of PP2A inhibitor proteins in other malignancies warrants further studies of cancer‐specific function for each of these proteins.

## Introduction

Colorectal cancer (CRC) is the third most common malignant neoplasm in many countries of the western world 1. Its prognosis has improved gradually as a result of advancements in surgery and adjuvant chemotherapy 2. However, a significant proportion of the patients still die of the disease 3. There is a need for biomarkers to predict which patients are at risk of disease recurrence. This would help to direct adjuvant treatments to those patients that gain benefit from them and protect the low‐risk patients from the side effects of therapy.

Protein phosphatase‐2A (PP2A) is a human tumor suppressor, which protects against cellular transformation. It is the major serine–threonine phosphatase, which functions by negatively regulating the activity of numerous signaling proteins important for malignant neoplasms 4, 5, 6. Consequently, the neoplasms have developed various mechanisms to oppose the PP2A activity 5, 7. Among these, three important endogenous PP2A inhibitors have been found: CIP2A, SET, and PME‐1 5, 7, 8, 9. Overexpression of CIP2A on protein level has been found in many human neoplasms, and it is a marker of poor outcome in several malignant neoplasms including CRC 10, 11. SET in turn seems to be particularly important in hematological malignancies 12. PME‐1 expression has been studied in only a limited number of human neoplasms, such as astrocytic gliomas, and endometrial, lung, and gastric cancers 9, 13, 14. Elevated amounts of PME‐1 have been found in endometrial and glial tumors, which are linked to the altered ERK pathway signaling, cell proliferation, and disease progression of gliomas to malignant subtypes 9, 13. Additionally, a small fraction (3–4%) of the gastric and lung cancer patients shows *PPME1* gene amplification, which also corresponds to elevated PME‐1 protein expression and activation of ERK and AKT survival signaling 14. These studies have highlighted potential oncogenic role of PME‐1 in these malignant neoplasms. However, whether oncogenic function of PME‐1 can be generalized to various human cancer types is as yet unclear. Also, the studies so far have failed to identify any correlation between tumor PME‐1 expression and the patient survival.

In this study, we report the immunohistochemical analysis of PME‐1 protein expression in the tumor material of a rectal cancer patient cohort, and its correlation to the clinicopathological parameters as well as patient survival. Unexpectedly, in strike contrast with its previously shown oncogenic role in other malignancies, we show that high PME‐1 expression correlates with superior clinical outcome in CRC. The association of high PME‐1 expression with better patient survival is confirmed at the mRNA level by using an independent CRC dataset. Finally, consistent with unexpected role for PME‐1 in CRC, PME‐1 inhibition in two human colon cancer cell lines fails to show any inhibitory effect on either cell survival or expression of phosphorylated AKT or ERK, shown to be regulated by PME‐1 in other previously studied cancer types.

## Materials and Methods

### Cell culture and siRNA transfections

Human colon carcinoma cell lines HCA‐7 and CW‐2 (gifted by Prof. Olli Carpén, University of Turku) were cultured in DMEM (Sigma‐Aldrich, Finland Oy, Helsinki, Finland) and RPMI (Sigma‐Aldrich) media, respectively, supplemented with 10% heat‐inactivated FBS (Gibco, Thermo Fisher Scientific Inc., Rockford, IL, USA), 2 mmol/L l‐glutamine, and penicillin (50 units/mL)–streptomycin (50 *μ*g/mL) in a humidified atmosphere of 5% CO_2_ at 37°C.

Small interfering RNA (siRNA) transfections were performed with Lipofectamine RNAiMAX reagent (Invitrogen, Life Technologies, Carlsbad, CA, USA) according to the manufacturer's instructions with the final siRNA concentration of 50 nmol/L per well. The scrambled (Scr) or control (5′‐GUA ACA AUG AGA GCA CGG C‐3′) and PME‐1‐specific (5′‐GGA AGU GAG UCU AUA AGC A‐3′) siRNAs were purchased from Eurofins MWG Synthesis GmbH, Germany. Three days after transfections, cells were harvested for analysis.

### Western blotting

Cells were lysed in 2× SDS sample buffer/Laemmli Buffer, boiled, and resolved by SDS‐PAGE. Proteins were transferred to PVDF membranes (Millipore, Merck KGaA, Darmstadt, Germany), which were blocked and incubated with required dilution of primary (at +4°C, incubated overnight) and 1:5000 dilution of secondary antibody (at room temperature, for 1 h) in 5% milk‐TBS‐Tween‐20, and developed by enhanced chemiluminescence (Pierce Biotechnology, Rockford, IL, USA). PME‐1, clone B‐12 (sc‐25278) antibody used at 1:1000 dilution, and phosphorylated AKT‐1/2/3 (Thr308) (sc‐16646) antibody used at 1:500 dilution were purchased from Santa Cruz Biotechnology Inc., Dallas, TX, USA. Antibody for phosphorylated ERK‐1/2 (Thr202/Tyr204) (#4370) used at 1:1000 dilution was purchased from Cell Signalling Technology, Danvers, MA, USA. Loading control antibody for GAPDH (5G4‐6C5) (1:200,000 dilution) was from HyTest Ltd., Turku, Finland. Densitometric analysis of the blots was performed using Image Lab software (Bio‐Rad Laboratories Inc., Hercules, CA, USA).

### Immunofluorescence

Cells were seeded on glass coverslips and transfected with siRNA. After 3 days, cells were fixed with 4% paraformaldehyde (Sigma‐Aldrich) and permeabilized with 0.5% Triton X‐100 (Sigma‐Aldrich) at room temperature for 10 and 5 min, respectively. Immunostainings were carried out with anti‐PME‐1 antibody (clone B‐12, sc‐25278) at 1:50 dilution in 10% goat serum blocking buffer, overnight at +4°C under constant rocking. Negative control coverslips were incubated with blocking buffer alone (without PME‐1 antibody). After 2–3 washes with PBS, coverslips were incubated with Alexa‐594‐conjugated goat anti‐mouse secondary antibody (A‐11005; Invitrogen, Life Technologies Ltd, Paisley, UK) for 1 h at room temperature. Nuclei were stained with Hoechst 33342 (Invitrogen), 1:2000 dilution in PBS for 10 min. Coverslips were mounted on glass slides over a drop of Mowiol (Sigma‐Aldrich), and images were acquired with AxioVert 200M fluorescence microscope (Carl‐Zeiss Microscopy GmbH, Göttingen, Germany) using 40× objective. Merged images were generated with ImageJ 15 (National Institutes of Health, Bethesda, MD).

### Cell viability assays

Cell viability was determined by two different assays; CellTiter‐glo (CTG) (Promega Corp., Madison, WI, USA) and cell proliferation reagent WST‐1 (Roche Diagnostics GmbH, Mannheim, Germany). CTG assay measures the cellular ATP levels as an indicator of metabolically active and viable cells. WST‐1 assay is dependent on NAD(P)H production by glycolysis and the activity of mitochondrial dehydrogenase enzymes, an indicator of metabolically active viable cells. Both the assays were performed as per the manufacturer's recommendations. CTG assay was performed in polystyrene 96‐well plates (Nunc, Thermo Fisher Scientific Inc., Paisley, UK) and luminescence was measured with Synergy H1 hybrid plate‐reader (BioTek, Winooski, VT, USA). The WST‐1 assay was performed in clear bottom 96‐well plates and the absorbance was measured at 450 nm.

### Tumor samples

Formalin‐fixed, paraffin‐embedded tumor samples were collected from patients treated for rectal cancer at Turku University Central Hospital between 2000 and 2009. These included operative samples (*n* = 210) with tumors of the middle and lower rectum from the archives of the Department of Pathology, Turku University Hospital. Superficial tumors operated by local excision were excluded from the study as well as patients with distant metastases at the time of diagnosis. The permission for using the archival tissue material was granted by the National Supervisory Authority for Welfare and Health, Finland (permission # Dnro 1709/32/300/02, 13 May 2002).

For tumor staging, we applied the sixth edition of TNM classification of malignant tumors in use at the time the patients were operated 16. Treatment was chosen according to preoperative tumor staging, including computerized tomography (CT) or magnetic resonance imaging (MRI) of the rectum, CT of the abdomen, and X‐ray or CT of the chest. Patients were treated either with short‐course preoperative radiotherapy (RT) (*n* = 88), long‐course preoperative (chemo) RT (*n* = 52), or received no treatment before surgery (*n* = 70) on the basis of common clinical recommendations 17. Short‐course RT was given 5 Gy fractions on 5 days, and the patients were operated on the following week. Long‐course RT was delivered in 1.8 Gy fractions to a total dose of 50.4 Gy in 6 weeks with (*n* = 43) or without (*n* = 9) chemotherapy. At that period, patients were operated 5–7 weeks after RT. The cytostatic treatment included either bolus 5‐fluorouracil (*n* = 4) or capecitabine (*n* = 39). The type of surgery was anterior resection among 113 (54%), and abdomino‐perineal resection among 93 (44%) patients. Four (2%) patients were operated with low Hartmann's procedure or other type of surgery. The majority of the specimens (*n* = 154) were screened to detect vascular invasion, which was found in 44 (28.6%) samples. Patients with established high‐risk features were treated with adjuvant chemotherapy. The median follow‐up time was 62.5 months. Disease recurrence was observed among 65 (31%) patients, either local or distant one. The clinical information of the patients is shown in Table 1.

**Table 1:** The clinical characteristics of the rectal cancer patients

	Total *n* = 210	Short‐course radiotherapy, *n* (%)	Long‐course radiotherapy, *n* (%)	Control, *n* (%)
Sex				
Male	119	54 (45)	32 (27)	33 (28)
Female	91	34 (37)	20 (22)	37 (41)
Mean age (years)		65	64	74
Preoperative T^a^				
T1–2	48	27 (56)	0 (0)	21 (44)
T3	68	54 (79)	2 (3)	12 (18)
T4	49	1 (2)	45 (92)	3 (6)
Tx	45	6 (13)	5 (11)	34 (76)
Postoperative T^a^^,^ b				
T1	10	3 (30)	2 (20)	5 (50)
T2	65	32 (49)	7 (11)	26 (40)
T3	111	49 (44)	26 (24)	36 (32)
T4	20	4 (20)	13 (65)	3 (15)
T0	4	0 (0)	4 (100)	0 (0)
Postoperative N^a^				
N0	124	51 (41)	34 (27)	39 (32)
N1	55	25 (46)	14 (25)	16 (29)
N2	28	12 (43)	4 (14)	12 (43)
Nx	3	0 (0)	0 (0)	3 (100)
Postoperative stage^a^				
Stage I	55	26 (47)	4 (7)	25 (46)
Stage II	66	25 (38)	24 (36)	17 (26)
Stage III	85	37 (44)	20 (23)	28 (33)
No viable tumor left	4	0 (0)	4 (100)	0 (0)
Postoperative grade^c^				
G1	32	9 (28)	10 (31)	13 (41)
G2	134	56 (42)	32 (24)	46 (34)
G3	35	21 (60)	3 (9)	11 (31)
Gx	9	2 (22)	7 (78)	0 (0)
Circumferential margin				
0	18	3 (17)	11 (61)	4 (22)
0≤ crm ≤2	21	8 (38)	6 (29)	7 (33)
>2	120	65 (54)	25 (21)	30 (25)
Unknown	51	12 (23)	10 (20)	29 (57)
Disease‐specific outcome				
Alive without recurrence	114	59 (52)	22 (19)	33 (29)
Alive with recurrence	9	3 (33)	3 (33)	3 (33)
Died of disease	56	16 (28)	20 (36)	20 (36)
Died of other causes	31	10 (32)	7 (23)	14 (45)

a T, the extent of tumor invasion; N, nodal status, and stage according to the TNM classification of malignant tumors 16.

b Includes the T3 tumors with threatened circumferential margin involvement.

c Postoperative tumor differentiation grade.

### Immunohistochemistry

Four of the 210 patients had no viable cancer cells left after preoperative treatment, classified as pT0. The amount of cancer cells was too scarce for a reliable evaluation of immunohistochemical staining in additional 11 patients. Consequently, samples of 195 patients were included in the final analysis. The most optimal paraffin blocks were selected to get enough tumor material for analyses. Sections of 5 *μ*m were cut. The antigen retrieval was performed with microwave oven twice for 7 min in 10 mmol/L sodium citrate buffer, pH 9. For immunohistochemical staining, monoclonal mouse‐anti‐human PME‐1 (clone B12) antibody epitope corresponding to amino acids 161–386 of PME‐1 of human origin (Santa Cruz Biotechnology, sc‐25278), at a dilution of 1:200 was used. For detection, the EnVisionTM + Dual Link System‐HRP (Dako, Glostrup, Denmark) was utilized.

### Analysis of PME‐1 expression

Two observes blinded to the clinical data evaluated the cytoplasmic and nuclear IHC staining of PME‐1 (A.E. all samples and E.M.B. 40 samples). Tissue from human glioblastoma was used as positive control. Both nuclear and cytoplasmic staining was scored on a scale of four intensity levels (+++, ++, +, −). The strong staining intensity (+++) corresponds to the positive control of PME‐1; weak staining intensity can still be distinguished from the background. Moderate staining intensity is intermediate between the previous ones. After estimating the predominant cytoplasmic and nuclear staining intensities, staining indices were analyzed. These include the most intensive cytoplasmic and nuclear indices. To calculate these indices, the area of the most intensive staining in cancer cells was chosen from each sample. After that, the fractions of cancer cells belonging to each staining intensity categories were estimated. The following formula was used to calculate the staining indices: I = 0*f0 + 1*f1 + 2*f2 + 3*f3, where I is the staining index and f0–f3 are the fractions of the cells showing a defined level of staining (from 0 to 3). Theoretically, the index can vary between 0 and 3 18.

### Evaluation of the tumor regression grade

Tumor regression grade (TRG) after long‐course RT was estimated by a pathologist (J.S.), using a scale of poor, moderate, or excellent TRG, according to a modified Dworak scale, as described previously 19. Briefly, poor TRG was defined as minimal or no tumor regression after (chemo) RT. In case of poor response, a considerable amount of tumor cells were still remaining after treatment. In tumors with moderate response, there were only some tumor cells or tumor cell groups left in the primary tumor (easy to find). In tumors with excellent response, very few or no tumor cells could be found.

### Statistical analysis

Statistical analyses for rectal cancer dataset were run using IBM SPSS Statistics 22.0.0.1 for Windows (IBM Corporation, Somers, NY) software package. Frequency tables were analyzed using the c2‐test, with the likelihood ratio (LR) or Fisher's exact test for categorical variables. Contingency tables (2 × 2) were used to calculate odds ratio (OR) and 95% confidence interval (CI) using the exact method. Fisher's exact test, Spearman's correlation, and LR were used to assess the significance of the correlation between individual variables in univariate analysis. Interobserver reproducibility of the assessments was tested with weighted kappa, calculated using the intraclass correlation coefficient (ICC) test, in parallel mode with a two‐way random model, using consistency assumption and the average‐measures option to interpret the ICC (95% CI). The ICC of assessments was very good, with weighted kappa values around 0.8.

Univariate survival analysis for disease‐free survival (DFS) and disease‐specific survival (DSS) was based on the Kaplan–Meier method where stratum‐specific outcomes were compared using log‐rank (Mantel–Cox) statistics. To adjust for the covariates, a Cox proportional hazards regression model was used. Covariates (as listed separately in results) were entered in a stepwise backward manner.

The TCGA colon and rectum adenocarcinoma (COADREAD) exon expression by RNAseq (Illumina HiSeq) dataset (*n* = 416) was downloaded from UCSC cancer genomics browser, and analyzed by JMP Pro 11.1.1 (SAS Institute Inc., Cary, NC, USA) software. *PPME1* expression distribution was studied and overall survival (OS) estimate curves were generated using Kaplan–Meier method (*n* = 396). Log‐rank chi‐square test was used to assess the significance of correlation between variables. To study the significance of *PPME1* mRNA expression compared to covariate variables, Cox proportional hazards regression model was fitted for the COADREAD dataset. Model fitting was done in R version 3.1.2 20 using package “Survival v.2.37‐7” 21. To make the results comparable, a set of covariates corresponding as closely to the clinical variables in the rectal cancer dataset as possible was aggregated from TCGA clinical data.

The statistical analyses of western blots was performed with MS excel using two‐tailed Student's paired *t*‐test. All statistical tests were two‐sided and declared significant at a *P*‐value of <0.05.

## Results

### Validation of the specificity of PME‐1 antibody in CRC cell lines

**Figure 1:**
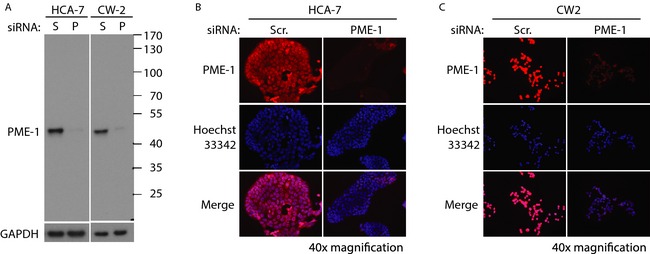
Validation of the specificity of PME‐1 antibody in colorectal cancer cell lines. (A) Western blot image of protein lysates from HCA‐7 and CW‐2 cells transfected with scrambled (S) or PME‐1 (P) siRNA (for 72 h), and blotted with PME‐1 (B‐12) antibody. GAPDH was used as a protein loading control. Black lines denote the location of protein molecular weight marker bands. Immunofluorescence images of HCA‐7 (B) and CW‐2 (C) cells transfected with Scr or PME‐1 siRNA (for 72 h), and incubated with PME‐1 antibody and visualized with anti‐mouse‐Alexa‐594 secondary antibody (red). Hoechst 33342 shows nuclear staining (blue). PME‐1 and nuclear staining overlay is shown in merge (fuchsia). All images were taken at 40× magnification.

In order to confirm the specificity of PME‐1 antibody to be used for immunohistochemistry of patient tumor material, we tested a PME‐1 antibody in colon cancer cell lines, HCA‐7, and CW‐2. PME‐1‐specific siRNA was used to knockdown its expression in these cells, which were lysed and subjected to western blotting with PME‐1 (clone B‐12) mouse monoclonal antibody (Fig. 1A). This antibody recognized only one band corresponding to the molecular weight of PME‐1 (44 kDa) in the control (Scr. siRNA transfected) cells. The intensity of this band was greatly reduced in the cells transfected with PME‐1 siRNA, suggesting that the antibody is specifically recognizing PME‐1 in these cell lysates. We used the same approach to test the PME‐1 antibody specificity by immunofluorescence, which corroborated the western blotting results in both HCA‐7 (Fig. 1B) and CW‐2 (Fig. 1C) cells. PME‐1 staining was present evenly in both cytoplasm and the nucleus in CW‐2 cells; however, the HCA‐7 cells displayed more intense staining in the nucleus as compared to the cytoplasm (Fig. 1B).

### PME1 protein expression in rectal cancer samples

**Figure 2:**
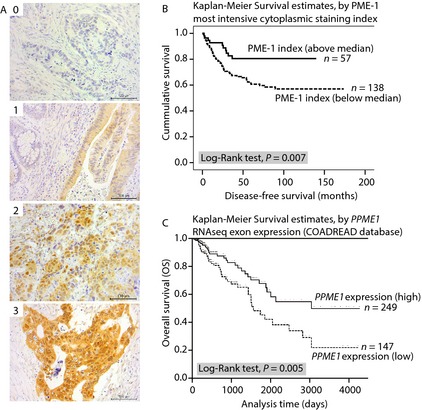
PME‐1 expression correlates with better survival of colorectal cancer patients. (A) Representative images of PME‐1 immunohistochemical staining from rectal carcinoma patient samples used for correlation analysis (scored 0–3). (B) Kaplan–Meier survival curve for disease‐free survival (DFS) (in months) analysis by PME‐1 most intensive cytoplasmic staining index in rectal cancer patient cohort (*n* = 195). (C) Kaplan–Meier survival curve for overall survival (OS) (in days) by *PPME1* gene expression (RNAseq exon array) in TCGA colon and rectal adenocarcinoma (COADREAD) patients (*n* = 396).

As observed in the colon cancer cell lines, the PME‐1 staining was localized both in the cytoplasm and nucleus, also in the sections made from paraffin blocks of clinical tumor samples. The staining intensity varied from one tumor to another, and the median of most intensive PME‐1 index concerning both cytoplasm and nucleus was 1.2. Normal rectal epithelium was mostly negative, although in some samples there was faint background‐like staining in some areas, especially in those near the cancer cells. Examples of negative, weak, moderate, and strong PME‐1 expression by IHC are illustrated in Figure 2A.

### PME‐1 protein expression related to clinicopathological variables

Among men, the most intensive cytoplasmic index of PME‐1 was more often below median than among women (*P* = 0.027, Pearson *χ* ^2^ test). The recurrent disease was more common among patients with most intensive cytoplasmic index of PME‐1 below median than those above median (*P* = 0.003, Pearson *χ* ^2^ test). The correlations of these clinicopathological variables and PME‐1 staining are shown in Table 2. There was no significant correlation between PME‐1 most intensive cytoplasmic staining index in relation to age, the nodal status, postoperative T, postoperative stage, postoperative grade, circumferential margin, vascular invasion, or postradiotherapy (RT) TRG (Table S1).

**Table 2:** Association of clinicopathological variables of rectal cancer patients with PME‐1 protein expression (most intensive cytoplasmic index)

Variable	Total *n* = 195^a^	PME‐1 most intensive cytoplasmic index		*P*‐value^b^
		Below median, *n* (%)	Above median, *n* (%)	
Sex				
Female	85	53 (62)	32 (38)	**0.027**
Male	110	85 (77)	25 (23)	
Recurrence				
Yes	57	49 (86)	8 (14)	**0.003**
No	138	89 (64)	49 (36)	

a PME‐1 most intensive cytoplasmic staining index could be analyzed from 195 patients only.

b Pearson chi‐square test.

### PME‐1 expression is related to DFS of rectal cancer patients

In the univariate survival analyses for the whole cohort, tumors with the most intensive cytoplasmic index of PME‐1 under median were linked to shorter DFS than those over median (110.7 vs. 116.7 months, *P* = 0.007, log‐rank test; Fig. 2B).

The multivariate (Cox) proportional hazards regression for DFS was performed for the patients with following covariates: treatment group, sex, age (70 years as cutoff), postoperative N (positive/negative), vascular invasion (positive/negative), circumferential margin (2 mm as cutoff), and the most intensive cytoplasmic index of PME‐1 (median as cutoff). The following remained as independent factors predicting a poor DFS of rectal cancer patients: sex if male (hazard ratio [HR] 4.12; 95% CI 0.25–0.98; *P* = 0.042), postoperative N (HR 10.33; 95% CI 1.68–8.40; *P* = 0.001), circumferential margin (HR 6.13; 95% CI 1.19–4.53; *P* = 0.013), and the most intensive cytoplasmic index of PME‐1 (HR 9.28; 95% CI 1.78–14.31; *P* = 0.002). The multivariate (Cox) proportional hazards regression for DSS was performed for patients with the similar covariates as that for DFS added with disease recurrence. The following remained as independent factors predicting a poor DSS: age if over 70 years (HR 4.46; 95% CI 0.24–0.95; *P* = 0.035) and disease recurrence (HR 21.62; 95% CI 15.50–840.54; *P* < 0.001). The multivariate (Cox) proportional hazards regression results for DFS and DSS have been presented in Table 3.

**Table 3:** Multivariate survival analysis of rectal cancer patient samples and TCGA colon and rectal adenocarcinoma (COADREAD) patient samples using Cox proportional hazards regression models

Variables	Rectal cancer (*n* = 142)^a^Disease‐free survival			Rectal cancer (*n* = 114)^a^Disease‐specific survival			TCGA COADREAD (*n* = 347)^a^Overall survival		
	HR	95% CI	*P*‐value	HR	95% CI	*P*‐value	HR	95% CI	*P*‐value
Sex									
Female	1			1			1		
Male	4.12	0.25–0.98	**0.042**	0.34	0.39–1.66	0.560	1.68	1.01–2.79	**0.046**
Age									
≤70 years	1			1			1		
>70 years	1.38	0.32–1.33	0.240	4.46	0.24–0.95	**0.035**	1.91	1.16–3.16	**0.011**
Circumferential margin									
≤2 mm	1			1					
>2 mm	6.13	1.19–4.53	**0.013**	1.63	0.78–3.20	0.201			
Postoperative N^b^									
Negative (N0)	1			1			1		
Positive (N1‐2)	10.33	1.68–8.40	**0.001**	0.79	0.54–4.99	0.374	3.15	1.79–5.53	**<0.001**
Vascular invasion^c^									
Negative	1			1			1		
Positive	1.93	0.82–3.16	0.165	0.42	0.36–1.66	0.519	1.47	0.86–2.48	0.162
Disease recurrence									
No				1					
Yes				21.62	15.50–840.54	**<0.001**			
PME‐1 expression^d^^,^ e									
High	1			1			1		
Low	9.28	1.78–14.31	**0.002** d	0.1	2.75–2.53	0.751^d^	2.22	1.32–3.72	**0.002** b

HR, hazard ratio; 95% CI, 95% confidence interval. Statistically significant *P*‐values are indicated in bold.

a Only 142 and 114 Rectal cancer patients for disease‐free survival and disease‐specific survival, respectively, and 347 TCGA COADREAD patients for overall survival could be analyzed for which complete data were available for all the covariates.

b Postoperative nodal status according to the TNM classification of malignant tumors 16.

c For TCGA data, vascular invasion indicates combined lymphatic, and/or venous invasion status.

d PME‐1 most intensive cytoplasmic index (protein expression) measured by IHC (cut‐off median).

e *PPME1* mRNA expression measured by RNA sequencing exon array Illumina HiSeq (cutoff −0.075).

### PME‐1 gene expression correlates with OS of CRC patients

In an independent colon and rectum adenocarcinoma (COADREAD, *n* = 396) RNA sequencing dataset (exon array IlluminaHiSeq) available from TCGA 22, we analyzed the correlation between OS and *PPME1* gene expression using UCSC Cancer Genomics Browser 23, 24. Although the PPME1 expression was normally distributed among this dataset, two distinct groups can be clearly isolated using cut‐off value of −0.075 (Figure S1A). Based on this cut‐off value, the data were categorized into two groups, low PPME1 (expression below −0.075) and high PPME1 (expression above −0.075) (Fig. 2C). This analysis revealed a similar trend at the mRNA expression level as was seen for PME‐1 protein expression in our rectal cancer dataset. The patient group with high PPME1 gene expression (*n* = 249) showed better OS (*P* = 0.005 log‐rank *χ* ^2^ test) than the patients with low PPME1 expression (*n* = 147). Similar results were seen using median PPME1 expression (0.07014) as a cutoff (Fig. 1B and C).

The multivariate analysis of TCGA COADREAD panel was performed by Cox proportional hazards regression models to assess the effect of other possible variables contributing to the prognosis of CRC patients in the Kaplan–Meier survival analysis. This analysis was carried out in 347 patients, for which full data was available for the following covariates: sex, age (cutoff 70 years), pathologic N (positive/negative), vascular invasion (positive/negative), and PPME1 gene expression (cutoff −0.075). This analysis revealed low PPME1 expression as an independent high‐risk factor (HR 2.22; 95% CI 1.32–3.72; *P*=0.002) predicting poor OS of CRC patients. Additionally, male gender (HR 1.68; 95% CI 1.01–2.79; *P* = 0.046), high age (>70 years) (HR 1.91; 95% CI 1.16–3.16; *P* = 0.011), and pathologic N positivity (HR 3.15; 95% CI 1.79–5.53; *P* < 0.001) were significant risk factors predicting poor OS of CRC patients. These results have been presented in Table 3. The multivariate analysis for TCGA COADREAD panel carried out using above mentioned covariates and median PME‐1 expression as cutoff also showed similar results (Table S2).

### Modulation of survival signaling by PME‐1 in CRC cells

**Figure 3:**
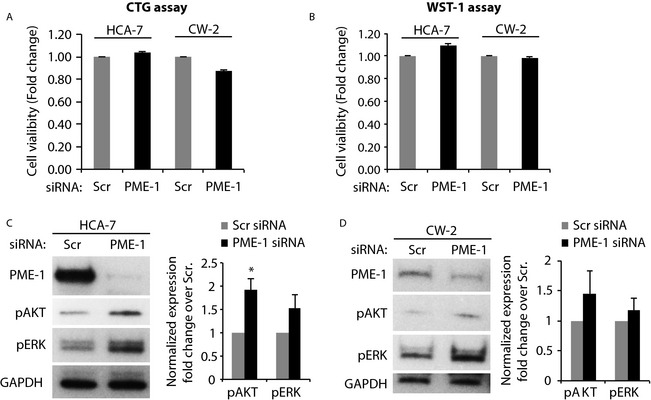
Modulation of survival signaling by PME‐1 in colorectal cancer cells. The viability of HCA‐7 and CW‐2 cells transfected with Scr. or PME‐1 siRNA (for 72 h) was analyzed by CellTiter‐glo (CTG) (A) and WST‐1 (B) assays. Bars represent fold‐change values calculated over respective values for Scr. siRNA‐transfected cells (*n* = 3). Western blot analysis of protein lysates from siRNA‐transfected HCA‐7 (C) and CW‐2 (D) cells, using antibodies to phosphorylated forms of AKT and ERK. The phosphoprotein expression normalized with GAPDH (loading control) is shown as fold‐change values over Scr. siRNA‐transfected cells (*n* = 3). *P = 0.003 by Student's paired *t*‐test.

PME‐1 silencing has been shown to inhibit viability and reduce the phosphorylated AKT and ERK levels in the glioblastoma cells 9 as well as in PME‐1 amplified gastric and lung cancer cells 14. To study the alterations in survival signaling by PME‐1 in colon cancer, PME‐1 specific siRNA was employed to knockdown its expression and the viability was analyzed in CW‐2 and HCA‐7 cell lines. Neither of the cell lines displayed significant difference in viability upon PME‐1 silencing, confirmed by using two different cell viability assays (Fig. 3A and B). Further immunoblotting analysis revealed that in contrast to other cancer types, PME‐1 silencing did not inhibit expression of active phosphorylated forms of serine–threonine‐specific protein kinases AKT‐1/2/3 (p‐AKT) and ERK‐1/2 (p‐ERK) in either of the colon cancer cell lines (Fig. 3C and D). In fact, the expression of p‐AKT in HCA‐7 cells was significantly higher (twofold, *P* = 0.003) in PME‐1 silenced cells as compared to the control (Scr. siRNA) cells (Fig. 3C); and even though the other effects did not reach the statistical significance, there was a clear trend toward higher p‐AKT also in CW‐2 cells and higher p‐ERK in both the cells lines upon PME‐1 inhibition. These findings demonstrate that opposite to other caner types PME‐1 may even decrease survival signaling in CRC cells and this may be linked to better patient outcome of rectal cancer patients with higher PME‐1 expression.

## Discussion

PME‐1 expression on protein level was studied in 195 rectal cancer patients. Among these patients, 52 were treated with long‐course (chemo) RT, 88 with short‐course RT, and 70 with surgery only. Our aim was to test PME‐1 as a potential marker to predict the outcome of rectal cancer patients.

There is little information available concerning the role of PME‐1 for patient outcome in human malignancies. Based on its molecular function as a PP2A inhibitor protein such as CIP2A 8, it would be expected that PME‐1 would also act as a human oncoprotein. It has been shown that PME‐1 is linked with the malignant progression of astrocytic gliomas 9 and endometrial cancers 13. For this reason it is surprising that PME‐1 protein expression seems to act in an opposite way in rectal cancer. High protein expression of PME‐1 is a marker for favorable outcome in univariate analysis of DFS for the whole cohort, and it remained an independent prognosticator of DFS together with sex, postoperative N status, and circumferential margin. Importantly, tumors with high PME‐1 protein expression were associated with fewer recurrences and a better disease outcome than those with low PME‐1 protein expression. The importance of these findings was further highlighted by the observation that in addition to the rectal cancer, colon cancer patients from an independent cohort (COADREAD) could be categorized into two groups based on *PPME1* mRNA expression, which correspond to differential OS outcome in both univariate and multivariate survival analyses. This information can be useful for establishing PCR tests based on *PPME1* expression as a biomarker to predict the survival of CRC patients 25, 26.

PME‐1 expression is known to be associated with increased cell proliferation and survival signaling in human malignant gliomas and endometrial cancers 9, 13. We studied these cellular functions in CRC cells. Since PP2A inhibition by PME‐1 has been previously shown to promote phosphorylation of the AKT and ERK proteins, it is surprising that in CRC cells these prosurvival signaling events are largely unaffected or affected in an opposite manner as compared to the other types of cancer. Mechanistically, PME‐1 promotes ERK pathway signaling mainly at a level upstream of Raf 9. However, a significant proportion of the CRCs contain activating mutations in K‐Ras (50%) and B‐Raf (10%), which promote ERK signaling independent of the upstream stimuli 27. In addition, the TGF*α*‐mediated autocrine feedback loop can further increase the Ras‐Raf‐ERK pathway signaling 27, 28. These alterations may render the CRC cells insensitive to PME‐1‐mediated regulation. Furthermore, inactivating mutations and/or altered expression of various PP2A components 29, 30, specifically the B‐subunits regulated by PME‐1, might affect the activity (phosphorylation) of specific target proteins. To this end, among the PP2A B‐subunits of PPP2R2 (B55) family, which are exclusively sensitive to PP2A C‐subunit Leu309 methylation reversibly removed by PME‐1 31, the PPP2R2B (B55*β*) subunit is epigenetically silenced by DNA hypermethylation in >90% of CRCs 32. The loss of this B‐subunit promotes drug resistance in CRC by activating the survival signaling in a PI3K‐AKT independent manner 32. It may be speculated that our surprising result of a favorable outcome with high PME‐1 expression is related to these altered pathways in CRC. Overall, the unexpected role for PME‐1 in CRCs is intriguing and calls for careful examination of cancer‐specific function for each of the PP2A inhibitor proteins when considering their role as biomarkers and potential targets for future cancer therapies.

## Conflict of Interest

None declared.

## Supporting information


**Figure S1.** PME‐1 mRNA expression correlates with colorectal cancer patient survival.

**Table S1.** Clinicopathological variables and the PME‐1 protein expression in rectal cancer patients.

**Table S2.** Multivariate survival analysis of TCGA colon and rectal adenocarcinoma (COADREAD) patients (*n* = 347) using Cox proportional hazards regression models.
