# Supplementary material for: Protein phosphatase methylesterase‐1 (PME‐1) expression predicts a favorable clinical outcome in colorectal cancer
Source: Cancer Med. 2015 Sep 17;4(12):1798–808. doi: 10.1002/cam4.541 (PMC5123709; doi:10.1002/cam4.541)
Supplement: Supplementary file 1 — Figure S1. PME‐1 mRNA expression correlates with colorectal cancer patient survival. [file CAM4-4-1798-s001.pdf]

Supplementary Figure 1

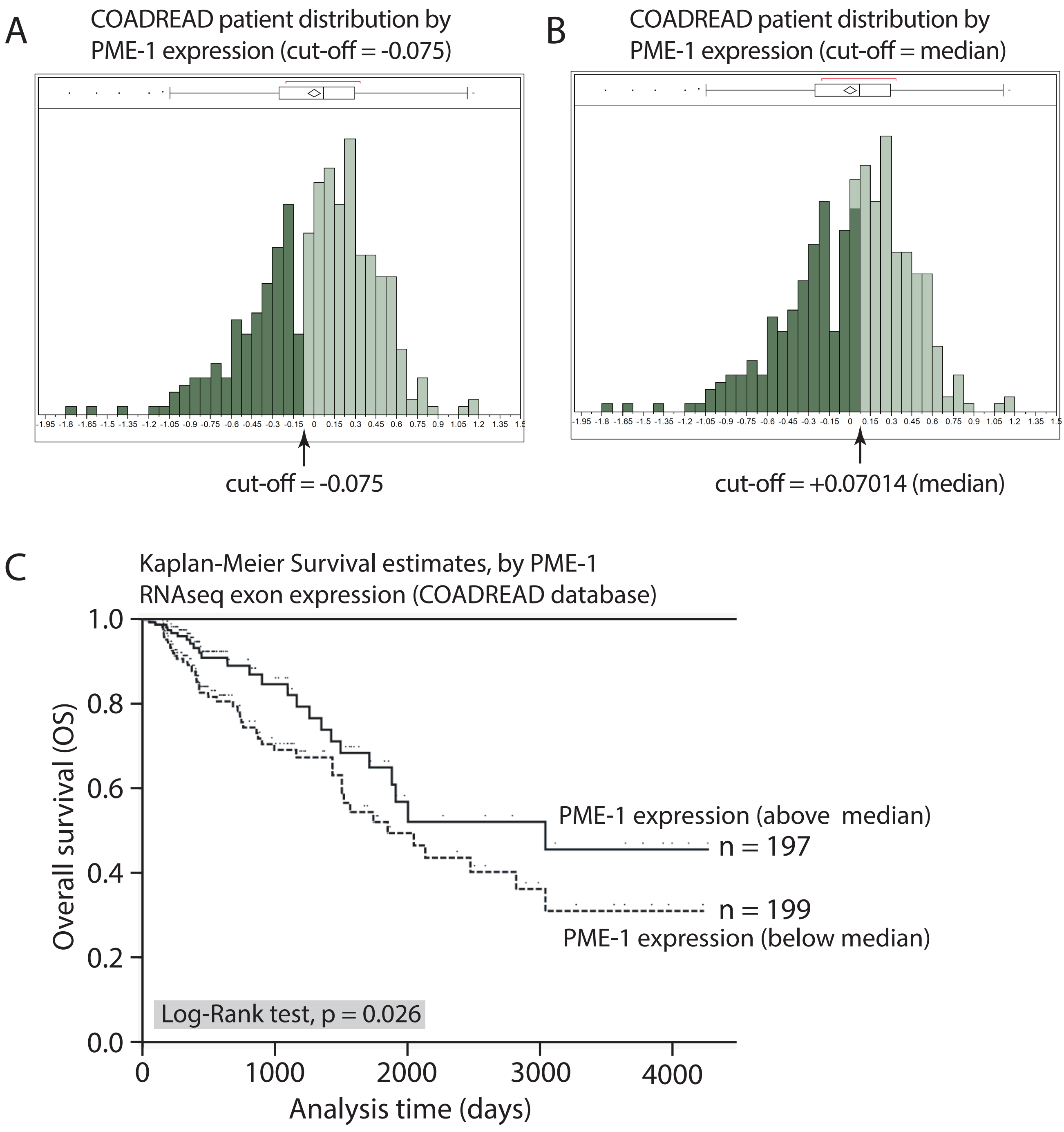

Supplementary Figure 1. PME-1 mRNA expression correlates with colorectal cancer patient survival. **(A and B)** Distribution plot for the TCGA colon and rectal adenocarcinoma patient (COADREAD) dataset based on the mRNA expression (RNAseq exon array) for PME-1. The patients categorized into two groups based on PME-1 expression are highlighted in different colour (dark green for low PME-1 and light green for high PME-1). Cut-off values of -0.075 **(A)** and +0.07014 or median **(B)** are marked with arrows. **(C)** Kaplan-Meier survival curve for OS by PME-1 gene expression in COADREAD patient groups based on median (+0.07014) PME-1 expression as cut-off (n = 396).
