# Supplementary material for: Protein phosphatase methylesterase‐1 (PME‐1) expression predicts a favorable clinical outcome in colorectal cancer
Source: Cancer Med. 2015 Sep 17;4(12):1798–808. doi: 10.1002/cam4.541 (PMC5123709; doi:10.1002/cam4.541)
Supplement: Supplementary file 2 — Table S1. Clinicopathological variables and the PME‐1 protein expression in rectal cancer patients. [file CAM4-4-1798-s002.doc]

**Supplementary Table 1.** Clinico-pathological variables and the PME-1 protein expression in rectal cancer patients.

| **Variable** |  | **PME-1 most intensive cytoplasmic index** | | **p-value** |
| --- | --- | --- | --- | --- |
|  | **Total** | **Below median, n** | **Above median, n** |  |
| ***Age***  Below 70 years  Above 70 years | 189 | 77  58 | 23  31 | 0.078 ^a^ |
| ***Postoperative N***  *N0*  *N1*  *N2*  *Nx* | 195 | 82  35  19  2 | 32  16  8  1 | 0.967 ^b^ |
| ***Postoperative T***  T1  T2  T3  T4  T0 (no vital cancer) | 195 | 5  46  70  17  0 | 1  16  37  2  1 | 0.086 ^b^ |
| ***Postoperative Stage***  I  II  III | 194 | 41  41  56 | 9  23  24 | 0.105 ^a^ |
| ***Postoperative Grade***  1  2  3  x | 195 | 25  85  25  3 | 3  44  8  2 | 0.055 ^b^ |
| ***Circumferential margin***  Under 2 mm  Over 2 mm | 153 | 24  81 | 16  32 | 0.504 ^a^ |
| ***Vascular invasion***  Yes  No  Cannot be assessed | 167 | 31  73  10 | 14  31  8 | 0.490 ^a^ |
| ***Postoperative TRG*** ^c^  Poor  Moderate  Excellent | 43 | 15  11  6 | 9  2  0 | 0.133 ^a^ |

^a^ Pearson Chi-square test; ^b^ Fisher's Exact test;

^c^ Patients treated with long-term (chemo) radiotherapy.
