# Supplementary material for: Protein phosphatase methylesterase‐1 (PME‐1) expression predicts a favorable clinical outcome in colorectal cancer
Source: Cancer Med. 2015 Sep 17;4(12):1798–808. doi: 10.1002/cam4.541 (PMC5123709; doi:10.1002/cam4.541)
Supplement: Supplementary file 3 — Table S2. Multivariate survival analysis of TCGA colon and rectal adenocarcinoma (COADREAD) patients (n = 347) using Cox proportional hazards regression models. [file CAM4-4-1798-s003.doc]

**Supplementary Table 2.** Multivariate survival analysis of TCGA colon and rectal adenocarcinoma (COADREAD) patients (n=347) using Cox proportional hazards regression models.

| **Variable** | **HR** | **95 % CI** | **p-value** |
| --- | --- | --- | --- |
| Male Gender | 1.75 | 1.05-2.91 | **0.032** |
| High age (>70 years) | 2.12 | 1.29-3.48 | **0.002** |
| Pathologic N (positivity) | 2.73 | 1.59-4.68 | **<0.001** |
| Vascular invasion ^a^ | 1.54 | 0.91-2.60 | 0.109 |
| Low PME-1 expression ^b^ | 1.87 | 1.12-3.13 | **0.016** |

HR, Hazard ratio; 95% CI, 95% confidence interval

^a^ Vascular invasion indicates combined lymphatic and/or venous invasion status.

^b^ PME-1 mRNA expression measured by RNA sequencing exon array IlluminaHiSeq (median as cut-off).
